# The Hospital and Nursing Exhibition

**Published:** 1924-05

**Authors:** 


### THE HOSPITAL EXHIBITION.

The annual Hospital, Nursing, and Midwifery Exhibition will open this year on Thursday, May 22, at the Central Hall, Westminster, and will continue through Friday and Saturday, May 23 and 24. There will be a large number of stands containing exhibits of interest to doctors, nurses, and other health workers, while the Hospital Section will devote special attention to matters of moment to hospitals from the constructional and equipment point of view. The fixture has grown in interest and importance from year to year, and now that its scope has been extended to the hospitals it bids fair to become an event of primary moment to all who are professionally concerned with the public health. Notices of the exhibits will be found elsewhere in this issue.

### The Hospital Officers' Conference.

This year there will be a new departure in connection with the Exhibition. As we announced last month, the first Annual Conference of the Incorporated Association of Hospital Officers is to be held during the three days of its duration under the presidency of Mr. H. L. Eason, C.B., C.M.G., M.D.

### Speakers and Subjects.

On the first day a paper will be given by Mr. H. J. Waring, F.R.C.S., Vice-Chancellor of London University (Senior Surgeon, St. Bartholomew's Hospital) on "Voluntary Hospitals and Paying Patients." In the afternoon, Mr. J. E. Stone, F.S.A.A. (Accountant to St. Thomas's Hospital), will read a paper on "Accountancy in Relation to Hospital Administration." In the evening the Annual Dinner will be held at the Holborn Restaurant.

### Railways and Hotels.

The Conference will resume at 10 a.m. on Friday the 23rd with a paper by Mr. A. E. Mason (Secretary, Midland Eye Hospital, Birmingham) on "The Changing Character of the Voluntary System." The chair will be taken by Viscount Burnham and Mr. A. H. Leaney (House Governor, the General Hospital, Birmingham) will lead the discussion. In the afternoon Mr. Frank G. Hazell (Gen. Supt. and Secretary, The Royal Infirmary, Manchester) will read a paper on "Maintenance." Visits will be paid to the various London Hospitals, the British Empire Exhibition, and other places of interest. Hotel accommodation has been reserved and the railway companies have arranged to grant special facilities to members attending the Conference, and on presentation of a voucher they will be able to obtain return rail tickets at the rate of single fares and one-third for the double journey.

The Association will have a stall in the Hospital Section of the Exhibition, adjoining which will be a small but attractive collection of old engravings and other views of hospitals. A special number of *The Hospital Gazette* will be published in connection with the Conference. The Hon. Secretary (Mr. J. E. Stone) will be pleased to forward particulars to all interested on application to 28 Bedford Square, W.C.1. It is hoped that succeeding Conferences will take place in the provinces during the next year or two, probably at Birmingham and Manchester.

